# Combined use of GM2AP and TCP1-*eta* urinary levels predicts recovery from intrinsic acute kidney injury

**DOI:** 10.1038/s41598-020-68398-0

**Published:** 2020-07-14

**Authors:** Víctor Blanco-Gozalo, Alfredo G. Casanova, Sandra M. Sancho-Martínez, Marta Prieto, Yaremi Quiros, Ana I. Morales, Carlos Martínez-Salgado, Consuelo Agüeros-Blanco, Adalberto Benito-Hernández, María A. Ramos-Barron, Carlos Gómez-Alamillo, Manuel Arias, Francisco J. López-Hernández

**Affiliations:** 1 Institute of Biomedical Research of Salamanca (IBSAL), Salamanca, Spain; 2 Department of Physiology and Pharmacology, University of Salamanca, Edificio Departamental, S-20, Campus Miguel de Unamuno, 37007 Salamanca, Spain; 3 Instituto de Estudios de Ciencias de La Salud de Castilla y León (IECSCYL), Soria, Spain; 4 Department of Nephrology, Hospital Universitario Marqués de Valdecilla, Instituto de Investigación Sanitaria Valdecilla (IDIVAL), Santander, Spain; 5 Group of Translational Research On Renal and Cardiovascular Diseases (TRECARD), Salamanca, Spain; 6 Group of Biomedical Research on Critical Care (BioCritic), Valladolid, Spain; 7 National Network for Kidney Research REDINREN, RD016/0009/0025, Instituto de Salud Carlos III, Madrid, Spain

**Keywords:** Biomarkers, Medical research, Nephrology, Kidney diseases

## Abstract

Deficient recovery from acute kidney injury (AKI) has immediate and long-term health, clinical and economic consequences. Pre-emptive recovery estimation may improve nephrology referral, optimize decision making, enrollment in trials, and provide key information for subsequent clinical handling and follow-up. For this purpose, new biomarkers are needed that predict outcome during the AKI episode. We hypothesized that damage pattern-specific biomarkers are expected to more closely associate to outcome within distinct subpopulations (i.e. those affected by specific pathological processes determining a specific outcome), as biomarker pleiotropy (i.e. associated to phenomena unrelated to AKI) introduced by unselected, heterogeneous populations may blur statistics. A panel of urinary biomarkers was measured in patients with AKI and their capacity to associate to normal or abnormal recovery was studied in the whole cohort or after sub-classification by AKI etiology, namely pre-renal and intrinsic AKI. A combination of urinary GM2AP and TCP1-*eta* best associates with recovery from AKI, specifically within the sub-population of renal AKI patients. This two-step strategy generates a multidimensional space in which patients with specific characteristics (i.e. renal AKI patients with good or bad prognosis) can be identified based on a collection of biomarkers working serially, applying pathophysiology-driven criteria to estimate AKI recovery, to facilitate pre-emptive and personalized handling.

## Introduction

Acute kidney injury (AKI) has a considerable and variable repercussion on patient’s health and health expenditure. AKI impact varies substantially, depending on the pathological scenario, patient characteristics, severity and type, but even mild^1–8^ and subclinical^9–14^ AKI have immediate and mediate consequences that increase general, cardiovascular and renal morbidity, and mortality, and the odds of progressing to chronic kidney disease (CKD)^15–17^. A distinctive case has to be made for critical patients, among whom AKI wreaks havoc, especially in the context of multiorgan failure. In the whole, AKI incidence within hospitalized patients ranges from 1 to 7%, and mortality reaches 23.9% in adults, and 13.8% in children^18^. Whilst in the intensive setting, incidence and mortality skyrocket, respectively, up to 30–50%^19,20^ and 40–80%^21–25^. Regardless of etiology, very severe AKI cases (i.e. those needing dialysis, AKI-D) hold worse prognosis^26^, which occur in 1–2% of hospitalized and 6–7% of critically ill patients^27^. The worst horizon is thus for critical patients with renal failure.

Defective recovery from AKI has critical repercussion on, and is thus a predictor of, short- and long-term morbidity and mortality^28^. Compared to non-AKI patients, AKI is associated to a higher mortality in the first 7 days, even after renal function normalization^29^. On the contrary, effective recovery is associated to lower risk of long term mortality and adverse renal complications^28,30^, which holds also true for AKI-D patients^31,32^. Defective recovery has been reported in the range of 11–53%^2,4,33,34^, depending on the study population and the definition of recovery. Persistent AKI may evolve to acute kidney disease (AKD) eventually leading to CKD^35,36^ in 19–31% of cases^15^, or even to sustained renal incompetence. For instance, 12.5% (1–64%, depending on the population) of patients require permanent dialysis after AKI^15^, a number that grows to 10–30% among AKI-D survivors^26^, and to 40–60% among those with prior CKD^37^. End stage renal disease caused by unsolved AKI increased from 1.2% of AKI cases by 1998 to 1.7% by 2003, and will continue to rise with the aging population and increase in comorbidities^15^. Recovery also impacts on expenditure. While AKI consumes 1% of total health budget^38^ and 5% of hospital expenditure^39,40^, slow recovery results in cost amplification ($2,600–7,933), derived from extended hospitalization (i.e. 3.9–5 extra days)^41–44^, additional monitoring and interventional procedures^39,45^.

Anticipating AKI outcome is a yet unmet challenge of clinical relevance, which would enable a closer monitoring and a personalized handling of patients with worse prognosis and slow or no recovery^45–47^, following international consensus protocols^36^. Outcome anticipation will also serve to better design clinical trials in order to target patients with poor prognosis^46^. Accordingly, identification of clinical predictors and biomarkers of recovery from AKI has been recognized among the key actions to reduce morbidity and mortality and to improve the quality of life of patients with more severe AKI^26^; and also among the top ten questions in the field of AKI research^46^. Unfortunately, current general severity scores (e.g. APACHE, SOFA) and AKI-specific severity scores are not good predictors of renal recovery^46^. Some studies have explored the association of biomarkers with longer-run outcomes [reviewed in^48^]. Specifically, urinary NGAL and HGF, and plasma IL18 and TNF receptor-1 were associated to 60-day, dialysis-free survival in dialysis-needing AKI patients. The TRIBE-AKI study examined the relation of urinary neutrophil gelatinase-associated lipocalin (NGAL), kidney injury molecule-1 (KIM-1), interleukin-18 (IL-18), liver-type fatty acid binding protein (L-FABP) and albumin with 3-year mortality after AKI, with uncertain conclusions. In the SAPPHIRE study, urinary tissue inhibitor of metalloproteinases-2 (TIMP-2) and insulin-like growth factor-binding protein 7 (IGFBP7) at intensive care unit admission were associated with death or dialysis at 9 months.

Prognosis and recovery are probably dictated by the underlying pathophysiological pattern resulting from each individual mixture of AKI etiologies (i.e. cause, type), and genetic and acquired determinants and comorbidities. Accordingly, new prognostic biomarkers are needed with defined pathophysiological meaning. In this article we hypothesized that damage pattern-specific biomarkers are expected to more closely associate to outcome within the subpopulation affected by such a pathological process. We found that a combination of the urinary levels of ganglioside GM2 activator protein (GM2AP) and chaperonin containing TCP-1, subunit eta (TCP1-*eta*) best associates to recovery from AKI, specifically within the sub-population of renal AKI patients.

## Results

Recovery from AKI was studied in a heterogeneous cohort of patients referred to the Nephrology Department (Hospital Universitario Marqués de Valdecilla, Santander, Spain) with AKI at call, and then followed for 30 days. As expected, individual evolution was very heterogeneous, ranging from patients recovering rapidly to others never reaching previous levels of renal function (i.e. Crpl) within the study timeframe. Urinary biomarkers of AKI were measured during the episode (early upon admission), and patients were associated to good or bad prognosis (i.e. complete or incomplete recovery within the following 30 days, respectively), regardless of AKI etiology. Controls were also included to provide the normal level range for each biomarker.

### Characteristics of control and AKI patients and urinary excretion of biomarkers

**Figure 1:**
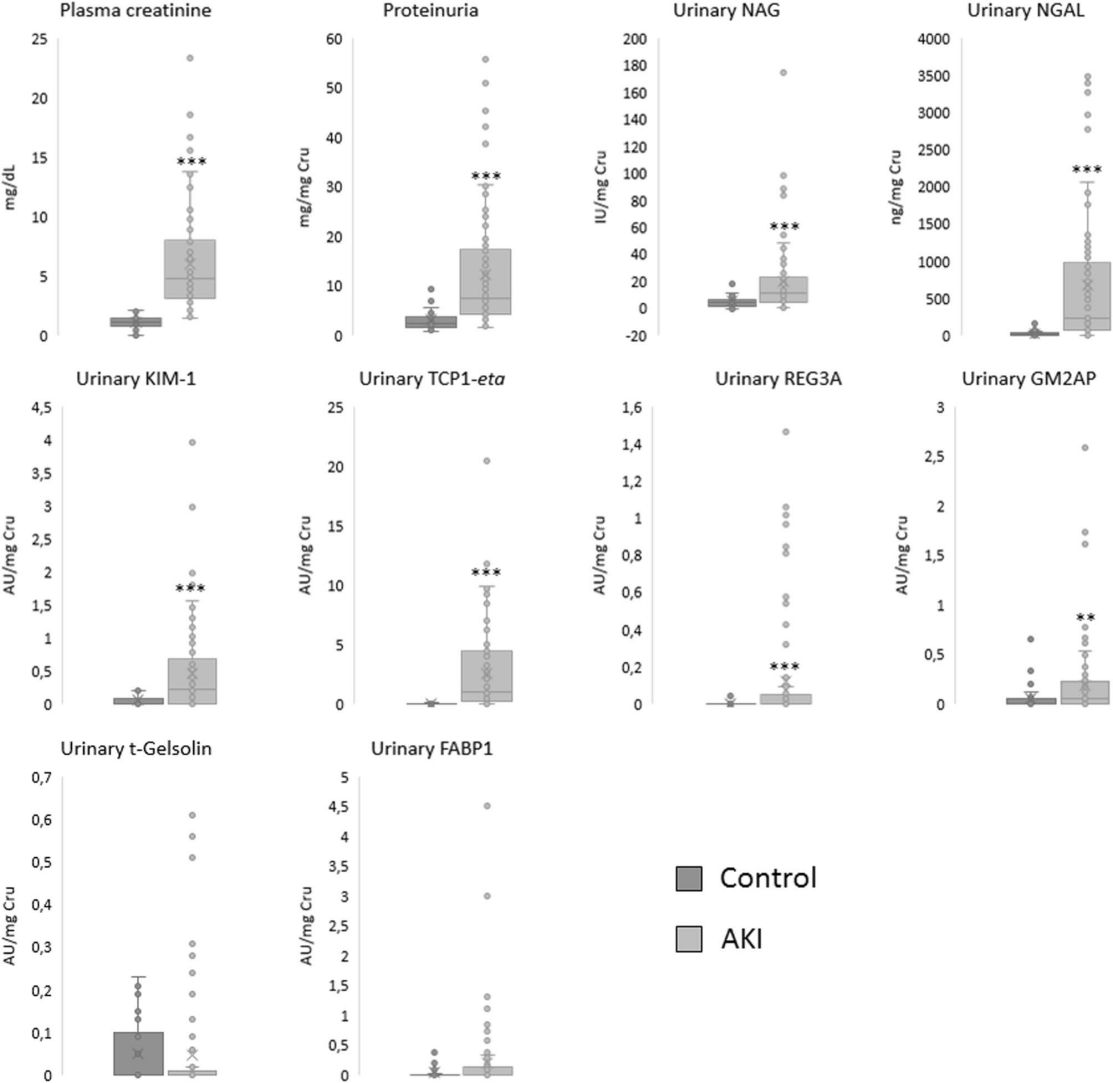
Biomarker levels in Control (n = 18) and AKI (n = 85) patients. *p < 0.05; **p < 0.01; ***p < 0.001 versus “Control” group. AU, arbitrary units; Cr_u_, urinary creatinine; FABP1: fatty acid binding protein 1; GM2AP, GM2-activator protein; KIM-1, kidney injury molecule 1; NAG, N-acetyl-β-d-glucosaminidase; NGAL, neutrophil gelatinase-associated lipocalin; REG3A, regenerating islet-derived 3 alpha; SD, Standard deviation; TCP1-*eta*, T-complex protein 1 *eta*.

Patient anthropometric and risk factor data are shown in Table 1. Plasma creatinine (Cr_p_), proteinuria and most of the urinary biomarkers (i.e. NAG, NGAL, KIM-1, TCP1-*eta*, Reg3A and GM2AP), except for t-gelsolin and FABP1, were significantly higher in patients with AKI (Fig. 1).

**Table 1:** Characteristics of the patients included in the study at call to Nephrology.

Patients included	AKI recovery (n = 43)	AKI non-recovery (n = 42)
Gender (male/female)	26/17	36/6**
	60.5%/39.5%	85.7%/14.3%
Age (years)	63.3 ± 2.3	63.9 ± 2.3
Weight (kg)	78.5 ± 2.4	77.5 ± 2.3
Height (cm)	167.4 ± 1.4	168.6 ± 1.6
Diabetes mellitus (no/yes)	33/10	25/17
	76.7%/23.3%	59.5%/40.5%
Hypertension (no/yes)	14/29	9/33
	32.6%/67.4%	21.4%/78.6%
Chronic kidney disease (no/yes)	32/11	22/20*
	74.4%/25.6%	52.4%/47.6%
Cardiovascular disease (no/yes)	21/22	16/26
	48.8% /51.2%	38.1%/61.9%
**KDIGO**		
0	1 (2.3%)	1 (2.5%)
1	7 (16.3%)	6 (15%)
2	6 (14%)	7 (17.5%)
3	29 (67.4%)	26 (65%)

Data are expressed as the absolute value (qualitative variables) or the mean (quantitative variables) ± standard error of the mean (SEM).

*AKI* acute kidney injury, *KDIGO* Kidney Disease: Improving Global Outcomes AKI classification.

*p < 0.05; **p < 0.01 versus the “recovery” group.

### Ability of the urinary biomarkers to predict recovery after an AKI episode

For further analysis, AKI patients were divided in two groups, namely those who subsequently recovered from AKI satisfactorily (i.e. Recovery), and those who did not (i.e. Non-recovery) during the following 30 days. Biomarkers measured at call to the Nephrology Department were then represented for Recovery and Non-recovery patients (Table 2a). Of note, the gold standard biomarker of AKI (i.e. Cr_p_) was not different between groups, which shows that Cr_p_ is neither informative nor related to recovery performance. However, significantly lower levels of GM2AP, FABP1, NGAL, t-gelsolin and REG3A were detected in the individuals who successfully recovered, compared to those individuals who did not recover. Among them, GM2AP showed the most statistically robust difference between groups. Similarly, a logistic regression analysis (Table 2b) confirmed that GM2AP hoards the greatest predictive capacity, being individually able to correctly discriminate the recovery pattern of 64.8% of patients. This analysis also showed that addition of TCP1-*eta* to the model increased the model’s success by 11.3%, to an overall 76.1%. TCP1-*eta* was revealed by the model as the biomarker best complementing GM2AP (Table 2c), despite showing no discrimination capability individually (Table 2a). Additional markers did not substantially improve prediction. Of note, NGAL performed very similarly to, but slightly less efficiently than TCP1-*eta*, when added to GM2AP.

**Table 2:** Biomarker levels in AKI patients

Recovery		Plasma creatinine (mg/dL)		Proteinuria (mg/mg Cr_u_)		Urinary NAG (IU/mg Cr_u_)		Urinary NGAL (mg/mg Cr_u_)		Urinary t-Gelsolin (AU/mg Cr_u_)		Urinary KIM-1 (AU/mg Cr_u_)		Urinary TCP1-*eta* (AU/mg Cr_u_)		Urinary REG3A (AU/mg Cr_u_)		Urinary FABP1 (AU/mg Cr_u_)		Urinary GM2AP (AU/mg Cr_u_)
**(a) Comparison between groups**																				
Yes (n = 43)	5.64 ± 0.56		11.43 ± 1.63		18.27 ± 4.98		480.67 ± 131.11		0.03 ± 0.01		0.44 ± 0.11		2.23 ± 0.61		0.09 ± 0.05		0.07 ± 0.02		0.15 ± 0.06	
No (n = 42)	6.39 ± 0.74		13.19 ± 2.04		21.43 ± 3.32		871.29 ± 156.91*		0.06 ± 0.02*		0.49 ± 0.11		2.89 ± 0.50		0.15 ± 0.04*		0.35 ± 0.13*		0.24 ± 0.06**	

Parameter	B	SD	Wald	*p* value
**(b) Logistic regression analysis (only GM2AP)**				
GM2AP	0.734	0.233	9.953	0.002
Constant	− 1.747	0.600	8.482	0.004
Total percentage of success: 64.8%				
Specificity: 69,4%				
Sensitivity: 60,0%				
**(c) Logistic regression analysis (GM2AP + TCP1-eta)**				
GM2AP	0.757	0.244	9.663	0.002
TCP1-*eta*	0.559	0.243	5.307	0.021
Constant	− 3.132	0.911	11.827	0.001
Total percentage of success: 76.1%				
Specificity: 75.0%				
Sensitivity: 77.1%				

(a) Concentrations of the different biomarkers according to subsequent recovery pattern. Data are expressed as the mean ± SEM. *p < 0.05; **p < 0.01; versus “Recovery—Yes” group. (b) Results of the logistic regression performed with GM2AP. c) Results of the logistic regression performed with GM2AP + TCP1-*eta*. AU: arbitrary units; Cr_u_: urinary creatinine; B: logistic regression coefficient; FABP1: fatty acid binding protein 1; GM2AP: GM2-activator protein; KIM-1: kidney injury molecule 1; NAG: N-acetyl-β-d-glucosaminidase; NGAL: Neutrophil gelatinase-associated lipocalin; REG3A: regenerating islet-derived 3 alpha; SD: Standard deviation; TCP1-*eta*: T-complex protein 1 *eta*; Wald: Wald statistic.

### Sub-stratification by parameters related to AKI etiology improves biomarker-mediated prediction of recovery

**Figure 2:**
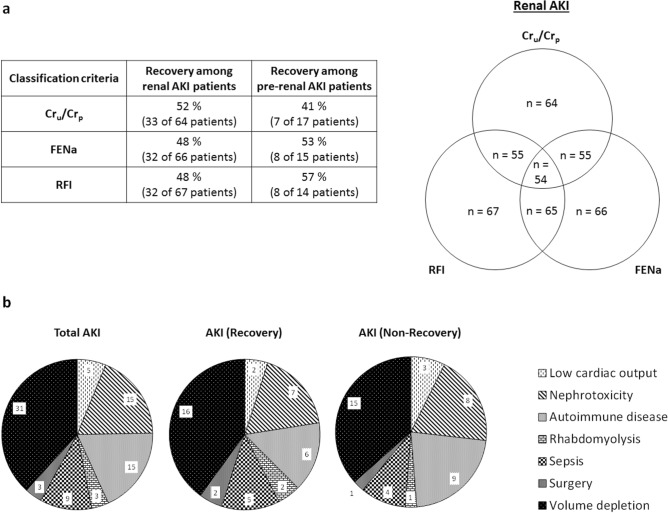
(**a**) Patient statistics according to the sub-classification criteria for pre-renal and renal AKI. Cr_p_, plasma creatinine; Cr_u_, urinary creatinine; FENa, fractional excretion of sodium; RFI, renal failure index. (**b**) AKI etiologies in the whole AKI population and in the subpopulation of patients who recovered from AKI and in the subpopulation of patients who did not recover.

**Figure 3:**
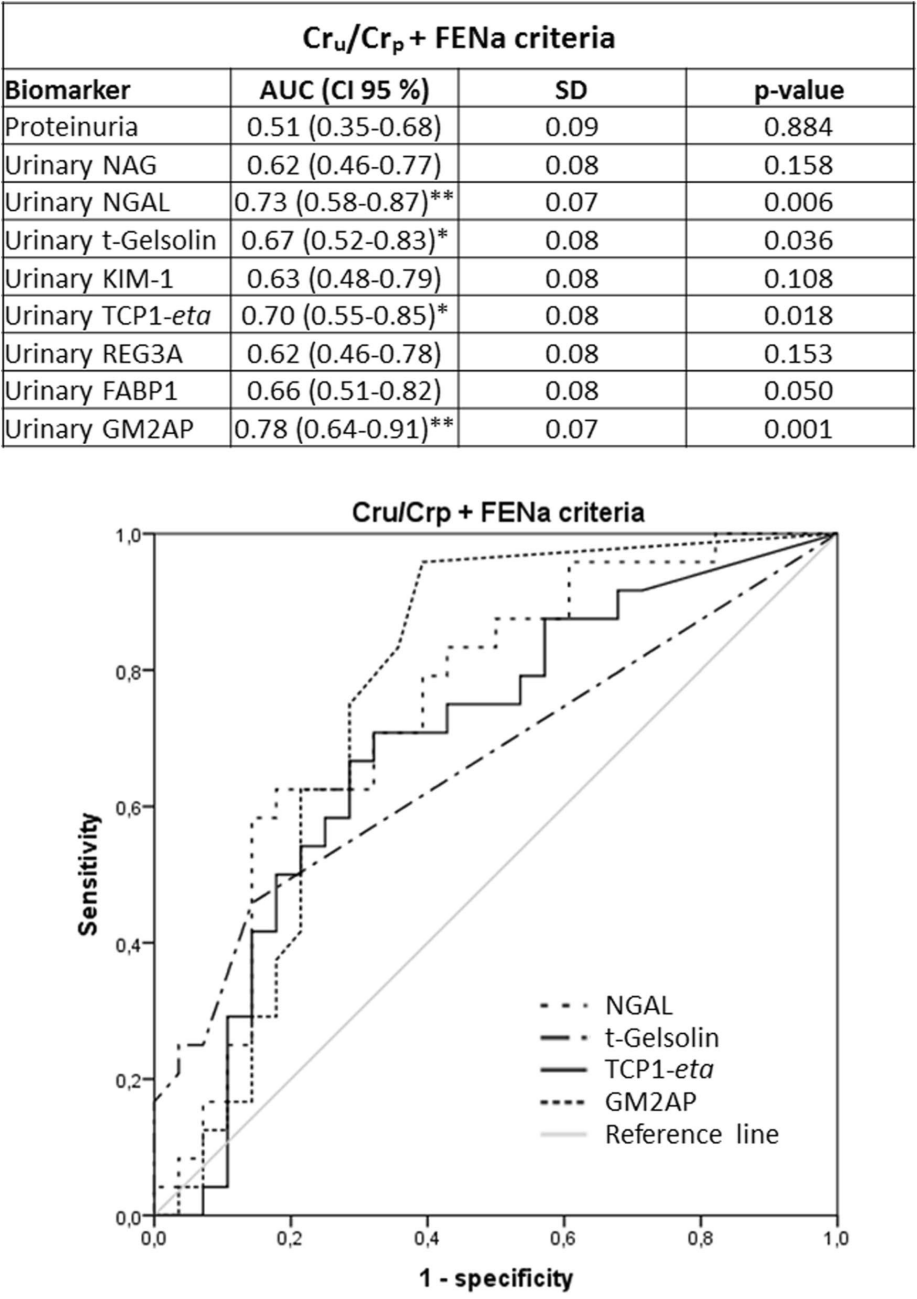
ROC curves of urinary biomarkers for intrinsic AKI patients sub stratified according to the double Cr_u_/Cr_p_ and FENa criteria (i.e. patients catalogued positive for intrinsic AKI complied with both criteria, n = 55). AUC: area under the curve; CI: confidence interval; Cr_p_: plasma creatinine; Cr_u_: urinary creatinine; FABP1: fatty acid binding protein 1; FENa, fractional excretion of sodium; GM2AP, GM2-activator protein; KIM-1, kidney injury molecule 1; NAG, N-acetyl-β-d-glucosaminidase; NGAL, neutrophil gelatinase-associated lipocalin; REG3A, regenerating islet-derived 3 alpha; RFI, renal failure index; SD, Standard deviation; TCP1-*eta*, T-complex protein 1 *eta*.

**Figure 4:**
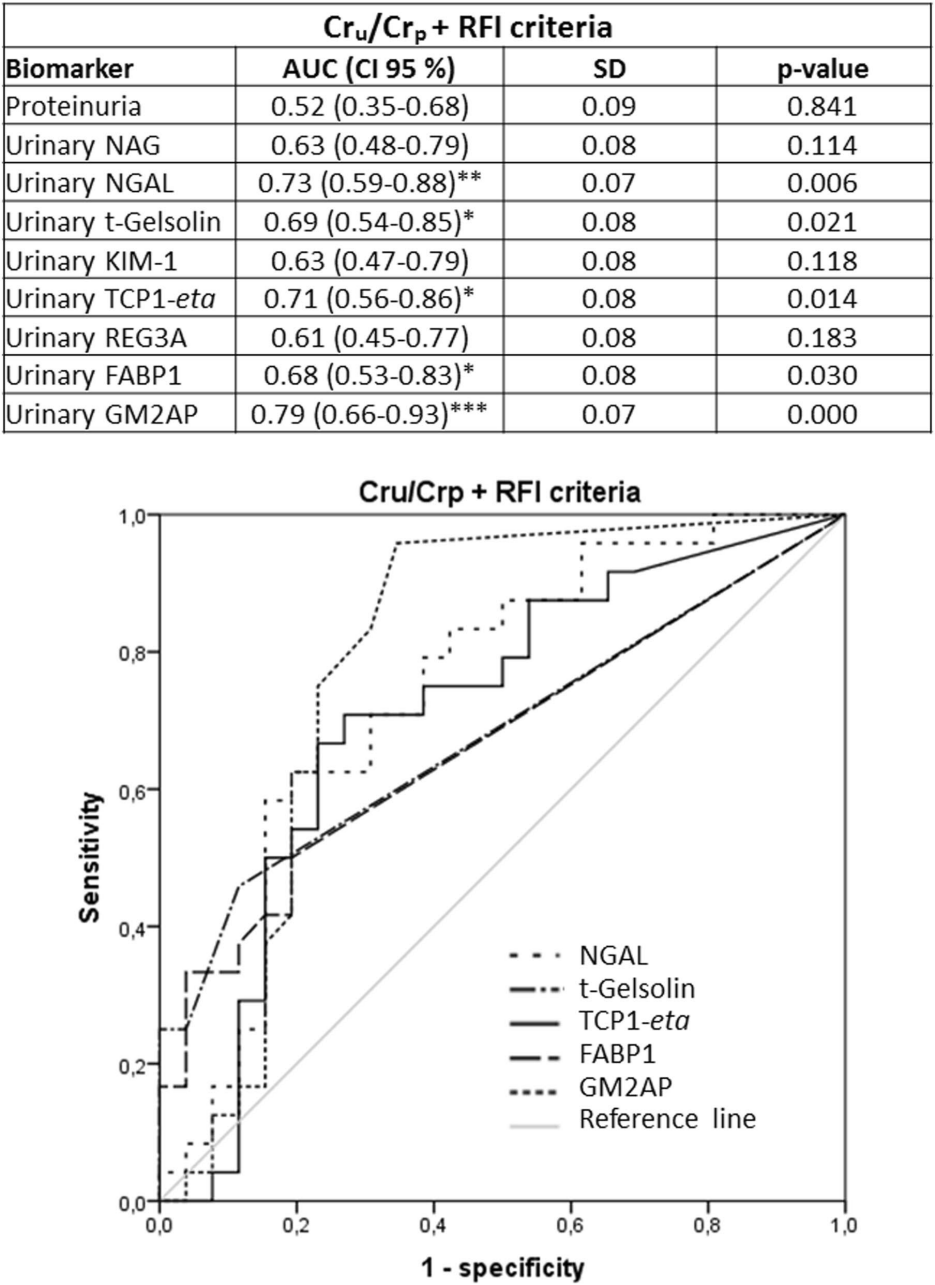
ROC curves of urinary biomarkers for intrinsic AKI patients sub stratified according to the double Cr_u_/Cr_p_ and RFI criteria (i.e. patients catalogued positive for intrinsic AKI complied with both criteria, n = 55). AUC: area under the curve; CI: confidence interval; Cr_p_: plasma creatinine; Cr_u_: urinary creatinine; FABP1: fatty acid binding protein 1; GM2AP, GM2-activator protein; KIM-1, kidney injury molecule 1; NAG, N-acetyl-β-d-glucosaminidase; NGAL, neutrophil gelatinase-associated lipocalin; REG3A, regenerating islet-derived 3 alpha; RFI, renal failure index; SD, Standard deviation; TCP1-*eta*, T-complex protein 1 *eta*.

**Figure 5:**
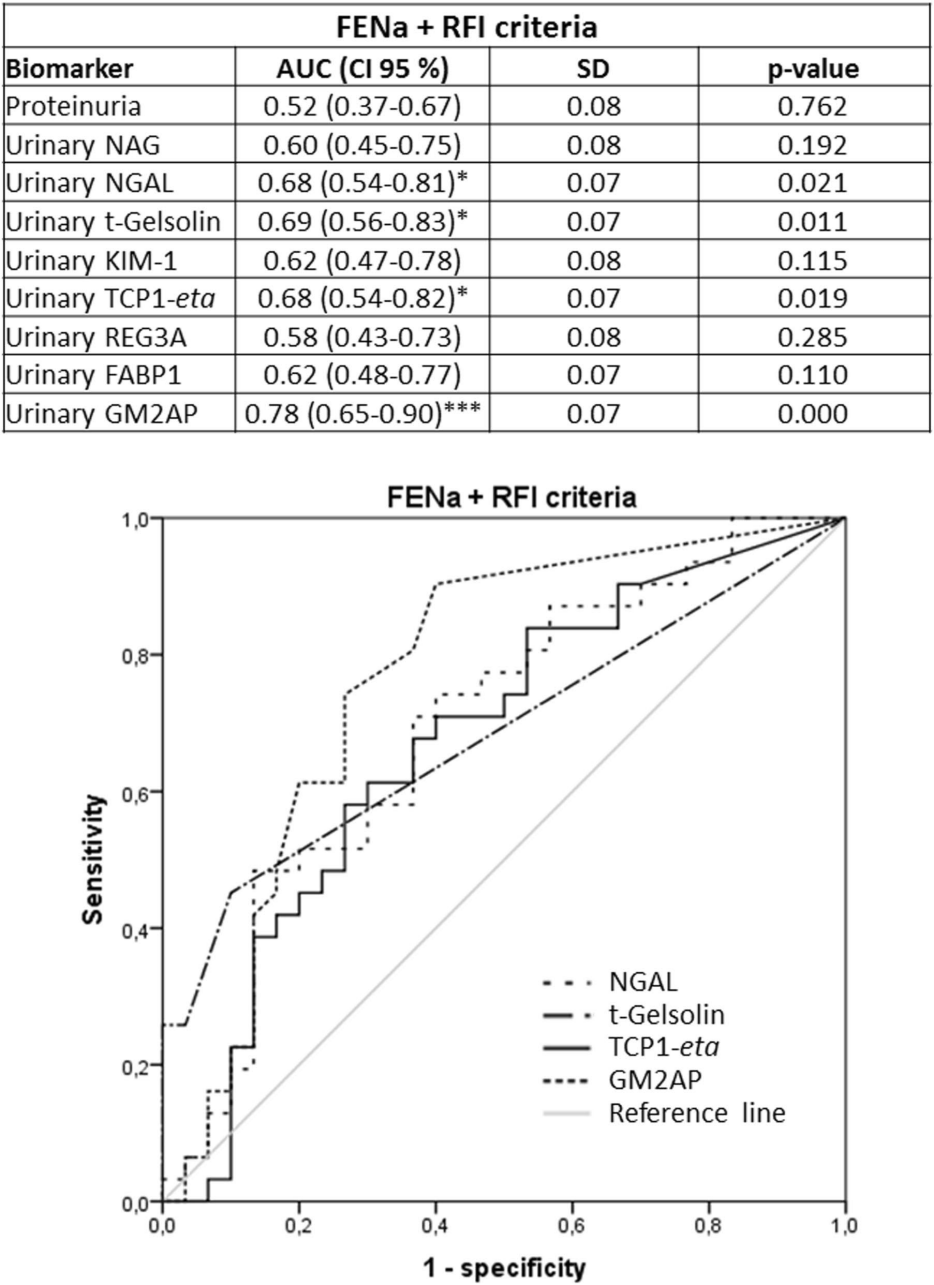
ROC curves of urinary biomarkers for intrinsic AKI patients sub stratified according to the double FENa and RFI criteria (i.e. patients catalogued positive for intrinsic AKI complied with both criteria, n = 65). AUC: area under the curve; CI: confidence interval; FABP1: fatty acid binding protein 1; FENa, fractional excretion of sodium; GM2AP, GM2-activator protein; KIM-1, kidney injury molecule 1; NAG, N-acetyl-β-d-glucosaminidase; NGAL, neutrophil gelatinase-associated lipocalin; REG3A, regenerating islet-derived 3 alpha; RFI, renal failure index; SD, Standard deviation; TCP1-*eta*, T-complex protein 1 *eta*.

**Figure 6:**
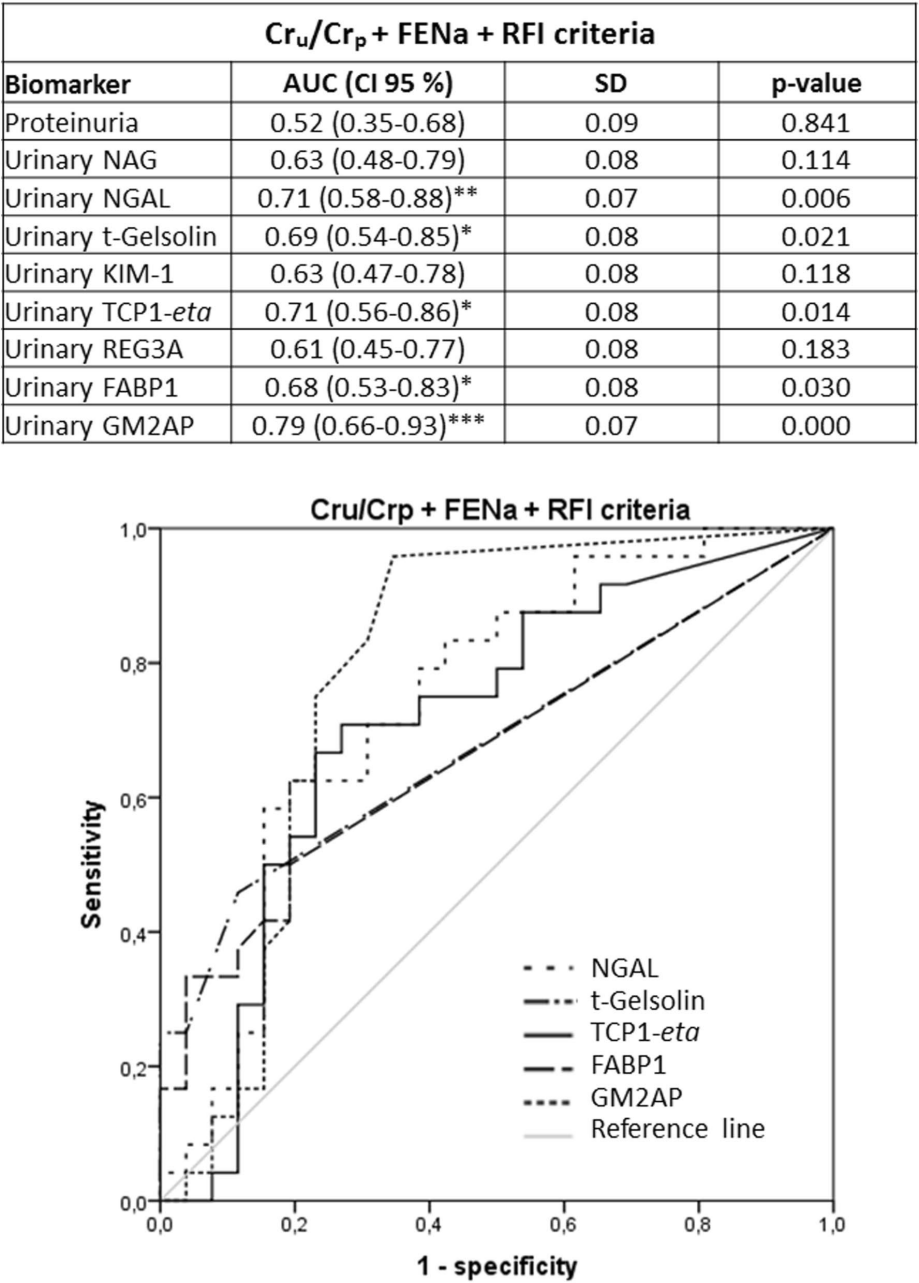
ROC curves of urinary biomarkers for intrinsic AKI patients sub stratified according to the triple Cr_u_/Cr_p_ and FENa and RFI criteria (i.e. patients catalogued positive for intrinsic AKI complied with all three criteria, n = 54). AUC: area under the curve; CI: confidence interval; Cr_p_: plasma creatinine; Cr_u_: urinary creatinine; FABP1: fatty acid binding protein 1; FENa, fractional excretion of sodium; GM2AP, GM2-activator protein; KIM-1, kidney injury molecule 1; NAG, N-acetyl-β-d-glucosaminidase; NGAL, neutrophil gelatinase-associated lipocalin; REG3A, regenerating islet-derived 3 alpha; RFI, renal failure index; SD, Standard deviation; TCP1-*eta*, T-complex protein 1 *eta*.

Previous studies had shown that the urinary biomarkers tested in this study were related to renal AKI [i.e. acute tubular necrosis (ATN)] [GM2AP^49^, TCP1-*eta*^50^, NGAL^6,51–53^, FABP1^53^, NAG^54,55^, t-gelsolin^56^ and REG3A^56^]. Accordingly, their capacity to predict AKI outcome (i.e. Recovery vs. Non-recovery) was further studied in the same cohort, now divided by markers associated with AKI etiology, specifically renal and pre-renal AKI. Sub-classification was made according to three criteria (namely Cr_u_/Cr_p_, FENa and the Renal Failure Index (RFI); see Material and Methods), considered individually and in double and triple combinations. The distribution of AKI patients into these sub-groups is shown in Fig. 2a, and their etiological description in Fig. 2b. Further etiological description of AKI patients who recovered from AKI and those who did not, within the pre-renal and renal AKI subpopulations is provided in Supplementary Figure 1. The number of renal AKI patients was very similar with all criteria. And most of these patients were captured by double and triple criteria. In fact, 54 patients of a total of 85 (63.53%) were considered to have had a renal AKI when the three criteria were used simultaneously. Furthermore, the % of patients recovering from the AKI was very similar regardless of the sub-classification criterion. Then, biomarker levels were redistributed according to the sub-classification groups (Tables 3, 4, 5). Importantly, in patients with biochemical characteristics of pre-renal AKI no biomarker showed a significant difference between Recovery and Non-recovery patients, with any of the sub-classification criteria. In contrast, within patients with biochemical characteristics of renal AKI (regardless of the classification criteria applied), significant higher levels of GM2AP (*p* < 0.001), NGAL, t-gelsolin (*p* < 0.01) and TCP1-*eta* (*p* < 0.05) were detected in the fraction of patients not recovering from AKI (i.e. Non-recovery). Based on these results, ROC curve analysis (Figs. 3, 4, 5, 6) and a logistic regression between biomarker level and outcome (Table 6) was performed in renal AKI patients. ROC curves showed that GM2AP had the highest AUC (*p* < 0.001), both when triple and double criteria combinations were used, although the AUC of NGAL, t-gelsolin and TCP1-*eta* are also noteworthy. Finally, logistic regression confirms that a combination of GM2AP and TCP1-*eta* generated the best predictive model with a success rate of around 80% regardless of the classification criteria used. In combination with GM2AP, NGAL substitutes TCP1-*eta* with very similar results, although again with slightly inferior performance. No association of outcome with etiology was evident in any of the cases (Supplementary Figure 1), which reinforces the utility of the new pathophysiological biomarkers.

**Table 3:** Urinary biomarker levels in pre-renal and renal-type AKI patients based on the Cr_u_/Cr_p_ criterion.

Recovery	Proteinuria (mg/mg Cr_u_)	Urinary NAG (IU/mg Cr_u_)	Urinary NGAL (mg/mg Cr_u_)	Urinary t-Gelsolin (AU/mg Cr_u_)	Urinary KIM-1 (AU/mg Cr_u_)	Urinary TCP1-*eta* (AU/mg Cr_u_)	Urinary REG3A (AU/mg Cr_u_)	Urinary FABP1 (AU/mg Cr_u_)	Urinary GM2AP (AU/mg Cr_u_)
**Pre-renal AKI patients: biomarker data as a function of recovery from AKI**									
Yes (n = 7)	10.23 ± 2.93	24.99 ± 15.06	197.54 ± 98.33	0.11 ± 0.07	0.44 ± 0.15	1.65 ± 1.38	0.00 ± 0.00	0.11 ± 0.05	0.08 ± 0.04
No (n = 10)	8.00 ± 1.66	16.61 ± 4.62	210.67 ± 99.91	0.07 ± 0.06	0.37 ± 0.15	1.97 ± 3.54	0.01 ± 0.01	0.02 ± 0.01	0.32 ± 0.16
**Renal AKI patients: biomarker data as a function of recovery from AKI**									
Yes (n = 33)	11.68 ± 1.89	17.05 ± 5.30	532.15 ± 152.68	0.00 ± 0.00	0.42 ± 0.13	2.42 ± 4.26	0.11 ± 0.06	0.06 ± 0.03	0.15 ± 0.08
No (n = 31)	14.92 ± 2.61	22.88 ± 4.09*	1,076.31 ± 188.17**	0.06 ± 0.02**	0.55 ± 0.14	3.30 ± 3.06*	0.20 ± 0.06*	0.42 ± 0.17*	0.21 ± 0.06*

Data are expressed as the mean ± SEM.

AU: arbitrary units; Cr_p_: plasma creatinine; Cr_u_: urinary creatinine; FABP1: fatty acid binding protein 1; GM2AP: GM2-activator protein; KIM-1: kidney injury molecule 1; NAG: N-acetyl-β-d-glucosaminidase; NGAL: Neutrophil gelatinase-associated lipocalin; REG3A: regenerating islet-derived 3 alpha; RFI: renal failure index; TCP1-*eta*: T-complex protein 1 *eta*.

*p < 0.05; **p < 0.01; ***p < 0.001 versus “Recovery: Yes” group.

**Table 4:** Urinary biomarker levels in pre-renal and renal-type AKI patients based on the FENa criterion.

Recovery	Proteinuria (mg/mg Cr_u_)	Urinary NAG (IU/mg Cr_u_)	Urinary NGAL (mg/mg Cr_u_)	Urinary t-Gelsolin (AU/mg Cr_u_)	Urinary KIM-1 (AU/mg Cr_u_)	Urinary TCP1-*eta* (AU/mg Cr_u_)	Urinary REG3A (AU/mg Cr_u_)	Urinary FABP1 (AU/mg Cr_u_)	Urinary GM2AP (AU/mg Cr_u_)
**Pre-renal AKI patients: biomarker data as a function of recovery from AKI**									
Yes (n = 8)	12.58 ± 2.47	24.37 ± 11.61	309.79 ± 136.34	0.08 ± 0.07	0.59 ± 0.19	4.75 ± 6.76	0.10 ± 0.10	0.02 ± 0.02	0.13 ± 0.05
No (n = 7)	12.80 ± 6.05	41.30 ± 13.05	193.62 ± 137.87	0.02 ± 0.01	0.20 ± 0.10	3.56 ± 5.08	0.15 ± 0.14	0.66 ± 0.64	0.16 ± 0.11
**Renal AKI patients: biomarker data as a function of recovery from AKI**									
Yes (n = 32)	11.14 ± 1.96	16.70 ± 5.57	524.78 ± 161.11	0.01 ± 0.01	0.38 ± 0.13	1.67 ± 2.64	0.09 ± 0.06	0.08 ± 0.03	0.14 ± 0.08
No (n = 34)	13.26 ± 2.20	18.51 ± 3.09	973.96 ± 172.96**	0.08 ± 0.03**	0.57 ± 0.13	2.83 ± 2.73*	0.15 ± 0.04	0.26 ± 0.10	0.25 ± 0.07***

Data are expressed as the mean ± SEM.

AU: arbitrary units; Cr_p_: plasma creatinine; Cr_u_: urinary creatinine; FABP1: fatty acid binding protein 1; GM2AP: GM2-activator protein; KIM-1: kidney injury molecule 1; NAG: N-acetyl-β-d-glucosaminidase; NGAL: Neutrophil gelatinase-associated lipocalin; REG3A: regenerating islet-derived 3 alpha; RFI: renal failure index; TCP1-*eta*: T-complex protein 1 *eta*.

*p < 0.05 **p < 0.01 ***p < 0.001 versus “Recovery: Yes” group.

**Table 5:** Urinary biomarker levels in pre-renal and renal-type AKI patients based on the RFI criterion.

Recovery	Proteinuria (mg/mg Cr_u_)	Urinary NAG (IU/mg Cr_u_)	Urinary NGAL (mg/mg Cr_u_)	Urinary t-Gelsolin (AU/mg Cr_u_)	Urinary KIM-1 (AU/mg Cr_u_)	Urinary TCP1-*eta* (AU/mg Cr_u_)	Urinary REG3A (AU/mg Cr_u_)	Urinary FABP1 (AU/mg Cr_u_)	Urinary GM2AP (AU/mg Cr_u_)
**Pre-renal AKI patients: biomarker data as a function of recovery from AKI**									
Yes (n = 8)	11.05 ± 1.75	15.11 ± 4.87	360.87 ± 141.56	0.02 ± 0.02	0.46 ± 0.19	4.81 ± 6.76	0.10 ± 0.10	0.10 ± 0.08	0.14 ± 0.05
No (n = 6)	12.80 ± 6.05	41.30 ± 13.05	193.62 ± 137.87	0.02 ± 0.01	0.24 ± 0.11	4.12 ± 5.32	0.18 ± 0.17	0.77 ± 0.75	0.18 ± 0.13
**Renal AKI patients: biomarker data as a function of recovery from AKI**									
Yes (n = 32)	11.52 ± 2.01	19.09 ± 6.17	511.59 ± 161.34	0.02 ± 0.02	0.41 ± 0.13	1.66 ± 2.62	0.09 ± 0.06	0.06 ± 0.03	0.14 ± 0.08
No (n = 35)	13.26 ± 2.20	18.51 ± 3.09	973.96 ± 172.96**	0.07 ± 0.03**	0.55 ± 0.12	2.75 ± 2.72*	0.15 ± 0.04*	0.25 ± 0.10	0.25 ± 0.07***

Data are expressed as the mean ± SEM.

AU: arbitrary units; Cr_p_: plasma creatinine; Cr_u_: urinary creatinine; FABP1: fatty acid binding protein 1; GM2AP: GM2-activator protein; KIM-1: kidney injury molecule 1; NAG: N-acetyl-β-d-glucosaminidase; NGAL: Neutrophil gelatinase-associated lipocalin; REG3A: regenerating islet-derived 3 alpha; RFI: renal failure index; TCP1-*eta*: T-complex protein 1 *eta*.

*p < 0.05; **p < 0.01; ***p < 0.001 versus “Recovery: Yes” group.

**Table 6:** Results of the logistic regressions performed with the biomarkers of patients with renal type AKI based on the different combinations of classification criteria used.

Parameter	B	SD	Wald	*p* value
**Cr**_**u**_**/Cr**_**p**_** + FENa + RFI criteria** **(n** = **54)**				
GM2AP	1.247	0.385	10.494	0.001
TCP1-*eta*	1.113	0.388	8.246	0.004
Constant	− 5.787	1.692	11.695	0.001
Total percentage of success: 82.7%				
**Cr**_**u**_**/Cr**_**p**_** + FENa criteria** **(n** = **55)**				
GM2AP	1.158	0.364	10.149	0.001
TCP1-*eta*	1.027	0.364	7.962	0.005
Constant	− 5.453	1.589	11.778	0.001
Total percentage of success: 71.7%				
**Cr**_**u**_**/Cr**_**p**_** + RFI criteria** **(n** = **55)**				
GM2AP	1.015	0.326	9.717	0.002
TCP1-*eta*	0.862	0.334	6.658	0.010
Constant	− 4.510	1.356	11.064	0.001
Total percentage of success: 81.1%				
**FENa + RFI criteria** **(n** = **65)**				
GM2AP	0.997	0.295	11.399	0.001
TCP1-*eta*	0.737	0.295	6.245	0.012
Constant	− 4.085	1.178	12.033	0.001
Total percentage of success: 77.8%				

B: logistic regression coefficient; Cr_p_: plasma creatinine; Cr_u_: urinary creatinine; FENa: fractional excretion of sodium; GM2AP: GM2-activator protein; RFI: renal failure index; SD: Standard deviation; TCP1-*eta*: T-complex protein 1 *eta*; Wald: Wald statistic.

## Discussion

The present study reveals that an algorithm combining the urinary levels of GM2AP and TCP1-*eta* during an AKI episode associates to subsequent prognosis, specifically to whether the patient will satisfactorily recover previous renal function or not. Prognosis works for the whole population of AKI patients in this study but, interestingly, its efficacy is strengthened for patients with biochemical characteristics of renal AKI, whereas it is lost among pre-renal AKI patients. This indicates that these biomarkers associate to specific pathophysiological events of renal AKI, and provide a potential diagnostic tool linking pathophysiology with outcome. This relation was previously observed in animal models, in which increased urinary excretion of these biomarkers was shown to be related to renal AKI^49,50^. GM2AP^49^ and TCP1-*eta*^50^ are urinary biomarkers associated to tubular damage, and to cortical tubular damage, respectively. GM2AP is an 18–24 kDa cofactor for the lysosomal β-hexosaminidase A implicated in GM2 ganglioside metabolism^57^, and in intercellular glycosphingolipid transport^58^. In the context of AKI, GM2AP increases in the urine as a result of defective tubular reabsorption of the filtered protein, subsequently to proximal tubular damage^56^ or sublethal alterations in proximal tubule transport^49^. TCP1-*eta* is a subunit forming a chaperonin-containing, hetero-oligomeric complex known to contribute to actin and tubulin folding, and thus to cytoskeleton conformation, cell shape^59^ and cell division^60^. As endoplasmic reticulum (ER) protein-folding chaperones, all TCP1 subunits are upregulated upon ER stress^61^, which links TCP1-*eta* to tubular damage. In fact, TCP1-*eta* is secreted by damaged cells, which contributes to its increased urinary excretion during AKI. Reduced tubular reclamation of the filtered TCP1-*eta*, associated to tubular damage, also contributes to its increased urinary level^50^. Interestingly, TCP1-*eta* urinary levels correlate with cortical damage level^50^.

AKI has been traditionally classified into three types, namely pre-renal, renal (or intrinsic) and post-renal^4,62–64^, with distinct etiopathology. In pre-renal AKI, kidney structures are preserved and, consequently, it is associated to a better clinical outcome than intrinsic AKI, which involves renal parenchymal damage^62,65–69^. The commonest pattern of intrinsic AKI is ATN, a rather ambiguous term comprising primary and heterogeneous damage forms to the renal tubular compartment^19,70^, including sublethal alterations in tubule cells compromising tubular function^71,72^. Prognosis of patients with no, mild or sub-lethal alterations may differ substantially from that of patients with extensive tissue destruction^70^. But this cannot be stablished until new diagnostic criteria and biomarkers become available to achieve the necessary degree of pathophysiological sub-classification. For example, despite pre-renal AKI being considered a mild form of AKI, our study reveals that Non-recovery patients split similarly between pre-renal and renal AKI patients, when they are etiologically triaged according to objective criteria.

In practice, etiopathological diagnosis of AKI and patient stratification have traditionally been poorly performed retrospectively, based on the duration of the episode and the response to fluid therapy^4,6,64,68,69^, rather than on unambiguous parameters. The gold standard biomarker (i.e. Cr_p_) conveys no etiological information as it increases in all forms of AKI^19,70^. In agreement, the present study shows that Cr_p_ has no association with the subsequent recovery pattern. Determining etiology is often further complicated by multi causality, as several potential causes of AKI frequently coexist and give rise to a variety of pathological combinations and damage patterns. In a number of pre-renal AKI cases damage evolves to a variable degree of intrinsic renal damage, as a complex continuum that further complicates diagnosis^6^ and patient triage. As such, outcome is not determined by ambiguous etiology, but by the resulting composite pathophysiological scenario. It is thus critical to sub stratify patients by AKI type, according to objective criteria before using pathophysiological biomarkers (e.g. GM2AP and TCP1-*eta*) for prognosis estimation. Our study utilizes a pathophysiology-driven and two-step strategy to further discriminate the prognostic utility of several AKI biomarkers to anticipate recovery. In a first, stratifying step the study population (i.e. all AKI patients) is narrowed to a more specific sub-population (renal AKI patients). In a second step, pathophysiological biomarkers are applied to this sub-population, through a combined algorithm, to estimate individual prognosis.

The prognostic capacity of other biomarkers, including KIM-1, L-FABP, IL-18, angiotensinogen, and TIMP-2/IGFBP7, has also been reported in the literature (reviewed in^73^), with individual prognostic AUCs around 0.8. However, comparisons with our study are difficult, as different outcome criteria, different populations and different methodology were used. Our best comparative opportunity is provided by our internal control, NGAL. NGAL has been shown to predict recovery, with higher urinary levels of NGAL associating to deficient or no recovery^74^. Interestingly, the composite prognostic capacity of GM2AP and TCP1-*eta* (as well as that of GM2AP alone) outperforms the predictive capacity of NGAL in our study. Because of biomarker pleiotropy and non-specificity, an appropriate algorithmic combination of several biomarkers related to the same phenomenon is more likely to discern populations than single biomarkers on their own, as the noise-to-signal ratio is expected to be lower^75,76^. This concept is supported by and congruent with our results. For instance, TCP1-*eta* and NGAL are not completely redundant with GM2AP, despite all being biomarkers of tubular damage^49,50,77^, because both markers increase the predictive capacity of GM2AP. Certainly, future studies will identify additional biomarkers that will further complement the algorithm to optimize prognosis.

In summary, the two-step strategy used in this study generates a multidimensional space in which patients with specific characteristics (in this case, renal AKI patients with good or bad prognosis) can be appropriately distinguished based on a collection of biomarkers that, working serially, focus the population (i.e. diagnostic stratification) and apply pathophysiology-driven criteria to estimate AKI recovery^19^, which will enable pre-emptive and personalized handling. In particular, the recovery pattern can be individually anticipated during AKI, by a model computing at least two criteria among Cr_u_/Cr_p_, RFI and FENa, plus the urinary level of both GM2AP and TCP1-*eta* at the moment of diagnosis.

## Materials and methods

Where not otherwise indicated, reagents were purchased from Sigma (Madrid, Spain).

### Patients and protocol

Urine samples were collected from 103 volunteers from the Nephrology Department (Hospital Universitario Marqués de Valdecilla, Santander, Spain), who provided written consent: 85 consultation patients referred to Nephrology had AKI at admission; and 18 controls, of whom 6 were disease controls (i.e. consultation patients without evidence of AKI), and 12 healthy individuals. All protocols were approved by the local Ethics Committee and were conducted according to the principles established in the Declaration of Helsinki (World Medical Assembly), the Council of Europe Convention on Human Rights and Biomedicine, the UNESCO Universal Declaration on the Human Genome and Human Rights, the requirements established in the Spanish legislation in the field of biomedical research, personal data protection and bioethics; as well as the provisions of the Law 14/2007, of July 3rd, of Biomedical Research; and RD 53/2013, of February1st.. Renal function and diagnosis data were obtained from the patients’ medical records. Renal function was monitored by means of Cr_p_, and AKI was defined and classified according to Kidney Disease: Improving Global Outcomes (KDIGO) criteria^78^. Urine was collected upon admission to the Nephrology Department, and was used to measure protein content (with a commercial kit from Bio-Rad, Madrid, Spain), and eight AKI-related biomarkers (as described below), namely NAG, NGAL, KIM-1, TCP1-eta, Reg3A, GM2AP, FABP1, and t-gelsolin. Patients were then followed for 30 days, and were then classified as Recovery or Non-recovery patients, depending on whether Cr_p_ had returned to basal levels ± 10% (i.e. the closest determination of Cr_p_ available in the medical record prior to the AKI episode) or not, respectively, in that period. Independently, patients were also classified as pre-renal and renal AKI based on three different criteria: (1) urinary creatinine/plasma creatinine ratio (Cr_u_/Cr_p_), (2) fractional excretion of sodium [FENa = (Na_u _× Cr_p_)/(Na_p _× Cr_u_) × 100] and (3) RFI = (Na_u _× Cr_p_)/Cr_u_. Individuals with a value > 20, < 1 or < 1, respectively, were classified as pre-renal^66,79–82^.

### Biomarker measurement by Western blot

21 μL of urine from each patient were separated by acrylamide electrophoresis. Proteins were transferred to an Immobilon-P Transfer Membrane (Millipore, Madrid, Spain) and incubated with the following primary antibodies: (1) Anti KIM-1 (R&D Systems, Minneapolis, MN, USA); (2) TCP1-eta antibody (Novus Biologicals, Littleton, CO, USA); (3) Reg3A (R&D Systems, Minneapolis, MN, USA); (4) GM2AP [our polyclonal antibody, described in^49^; (5) gelsolin (Santa Cruz Biotechnology, Dallas, TX, USA); and (6) FABP1 (SAB Signalway Antibody, College Park, MD, USA). Then, membranes were incubated with horseradish peroxidase-conjugated secondary antibodies and chemiluminescent detection (Immobilon Western Chemiluminescent HRP Substrate kit; Millipore, Madrid, Spain) with photographic films (Kodak, Madrid, Spain). Bands were quantified with the Scion Image software (Scion Corporation, Frederick, Maryland, USA), and normalized to the signal of a positive control (as arbitrary units), loaded in all gels. The positive control consisted of a urine sample from a designated AKI patient with increased biomarker excretion, used as trans normalization control in all experiments.

### NAG determination

NAG activity was quantified using a commercial kit [N-Acetyl-β-d-glucosaminidase (NAG) assay kit, Diazyme, Poway, CA, USA] following the manufacturer's instructions.

### NGAL determination

NGAL was measured with a commercial ELISA (Human NGAL ELISA Kit 036CE (BioPorto Diagnostics, Hellerup, Denmark), according to the manufacturer’s instructions.

### Statistical analysis

Data are expressed as mean ± SEM (except where indicated otherwise). The Kolmogorov–Smirnov test was used to evaluate if the numerical data were adjusted to a normal distribution (*p* values < 0.05 were considered non-normal). Comparisons of urinary biomarkers between Control versus AKI and Pre-renal versus Renal patients were performed using Student's t test (for normal data) or Mann–Whitney U test (for non-normal data), in which *p* values < 0.05 were considered statistically different. To assess the ability of the markers to identify whether or not a patient will recover from the AKI, ROC curves were drawn from each of them. Their areas under the curve (AUC) were calculated and compared with that of a hypothetical marker with zero diagnostic capacity (AUC = 0.50). *p* values < 0.05 were considered statistically significant^83^. In order to establish if any of the biomarkers or some combination of them was able to predict mathematically the probability of a patient to recover or not from the AKI, a binary logistic regression was performed in which *p* values < 0.05 were considered statistically significant (the biomarker has predictive capacity). The construction of the ROC curves and the logistic regression model was carried out after distributing the urinary excretion of each biomarker in quartiles (Q1: low excretion, Q2: medium–low excretion, Q3: medium–high excretion and Q4: high excretion)^52^. All the statistical studies described were performed with the IBM SPSS Statistics 20 software (IBM, Armonk, NY, USA). The tables and figures were created with the IBM SPSS Statistics 20 and Microsoft Excel 2016 (Microsoft, Redmond, WA, USA).

## Supplementary information

Supplementary Legends.

Supplementary Figure 1.
