# Supplementary material for: Combined use of GM2AP and TCP1-eta urinary levels predicts recovery from intrinsic acute kidney injury
Source: Sci Rep. 2020 Jul 14;10:11599. doi: 10.1038/s41598-020-68398-0 (PMC7360779; doi:10.1038/s41598-020-68398-0)
Supplement: Supplementary file 1 — Supplementary Legends. [file 41598_2020_68398_MOESM1_ESM.docx]

**Supplementary Figure 1.** AKI etiologies in AKI patients who recovered from AKI and those who did not, within the pre-renal and renal AKI subpopulations according to the three sub classification criteria, and by the three possible combinations of two of them. Cr_p_, plasma creatinine concentration. Cr_u_, urinary creatinine concentration. FENa, fractional excretion of sodium. RFI, renal failure index.
