# Supplementary figures and images for: Combined use of GM2AP and TCP1-eta urinary levels predicts recovery from intrinsic acute kidney injury
Source: Sci Rep. 2020 Jul 14;10:11599. doi: 10.1038/s41598-020-68398-0 (PMC7360779; doi:10.1038/s41598-020-68398-0)

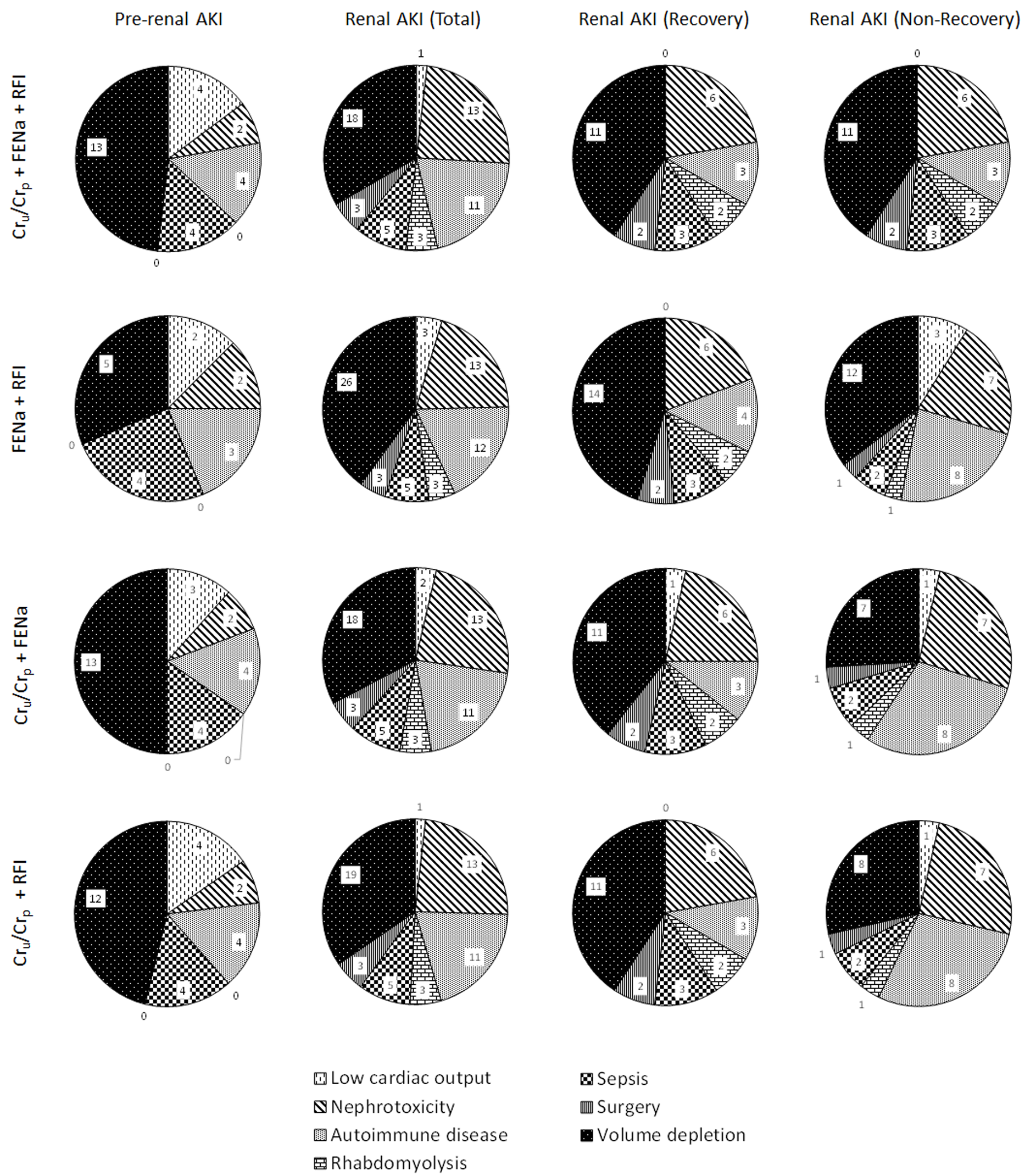

Supplement: Supplementary file 2 — Supplementary Figure 1. [file 41598_2020_68398_MOESM2_ESM.tif]
